# Arbuscular Mycorrhizal Fungi Confer Salt Tolerance in Giant Reed (*Arundo donax* L.) Plants Grown Under Low Phosphorus by Reducing Leaf Na^+^ Concentration and Improving Phosphorus Use Efficiency

**DOI:** 10.3389/fpls.2019.00843

**Published:** 2019-07-16

**Authors:** Antònia Romero-Munar, Elena Baraza, Javier Gulías, Catalina Cabot

**Affiliations:** ^1^Research Group on Plant Biology Under Mediterranean Conditions, Department of Biology, Facultat de Ciències, University of the Balearic Islands, Palma, Spain; ^2^Institute of Agro-Environmental and Water Economy Research (INAGEA), Palma, Spain

**Keywords:** early stage, arbuscular mycorrhiza, salinity tolerance, phosphorus scarcity, phosphorus use effieciency

## Abstract

Salinization is one of the major causes of agricultural soil degradation worldwide. In arid and semi-arid regions with calcareous soils, phosphorus (P) deficiency further worsens the quality of salinized soils. Nonetheless, nutrient poor soils could be suitable of producing second-generation energy crops. Due to its high biomass production, *Arundo donax* L. (giant reed) is one of the most promising species for energy and second-generation biofuel production. *A. donax* can be propagated by micropropagation, an *in vitro* technique that produces high number of homogeneous plantlets. However, crop establishment is often compromised due to poor plantlet acclimatization to the soil environment. Arbuscular mycorrhizal fungi (AM) are components of soil-plant systems able to increase root phosphorus uptake and to confer the plant an increase tolerance to salinity with a consequent enhancement effect of plant growth and yield. In the present study, the relative importance of the early symbiosis establishment between AM fungi and *A. donax* micropropagated plantlets in the response to salt stress under low phosphorus availability was determined. A commercial inoculum which contained two different AM fungi species: *Rhizophagus intraradices* and *Funneliformis mosseae* was used. AM-symbionts (AM) and non-symbionts plants were grown at two phosphorus [2.5 μM (C) and 0.5 mM (P)] and three NaCl (1, 75 and 150 mM) concentrations in a room chamber under controlled conditions. After 5 weeks, AM root colonization was 60, 26 and 15% in 1, 75 and 150 mM NaCl-treated plants, respectively. At 1 and 75 mM NaCl, AM plants showed increased growth. In all saline treatments, AM plants had decreased Na^+^ uptake, Na^+^ root-to-shoot translocation, Na^+^/K^+^ ratio and increased P and K use efficiencies with respect to C and P plants. AM improved the nutritional status of *A. donax* plants by enhancing nutrient use efficiency rather than nutrient uptake. Increased phosphorus use efficiency in AM plants could have benefited ion (Na^+^ and K^+^) uptake and/or allocation and ultimately ameliorate the plant’s response to saline conditions.

## Introduction

Salinity is one of the most damaging degradation processes affecting soils, especially in arid and semi-arid regions, where salinization is considered a major cause of soil desertification. According to Dubois (2011), salinity affects 19.5% of irrigated and 2.1% of dry agricultural lands worldwide.

Soil salinization negatively affects plant growth and yield. The high salt concentration in the soil solution decreases the soil osmotic potential that may result in loss of cell turgor in species unable to regulate their water potential. Additionally, the excess of ions, principally Na^+^ and Cl^–^, negatively affect plant metabolism by inducing ion toxicity or/and ion imbalance in plant tissues (Marschner, 2011). Nonetheless, plants have evolved multiple responses to cope with salt stress. Control of water and ion homeostasis, Na^+^ exclusion from the shoot, Na^+^ tissue tolerance and the scavenging of toxic compounds are among the principal physiological and biochemical mechanisms involved (Hasegawa et al., 2000; Munns and Tester, 2008).

On the other hand, in the Mediterranean climatic regions, where calcareous and alkaline soils largely prevail (Vance et al., 2003), in addition to salinity, crop production is also threated by P deficiency, especially in low-input agricultural systems. Changes in growth and root structure (Lambers et al., 2006) and increased synthesis and secretion of phosphatase into the rhizosphere to improve soil P mobilization (Li et al., 2011) are common plant responses to cope with P scarcity. The exudation and increased root levels of strigolactones, compounds that trigger mechanisms involved in the plant- Arbuscular Mycorrhizal (AM) fungi interaction, are also promoted by P deficiency (Akiyama et al., 2005; Yoneyama et al., 2007).

In natural habitats, plants often simultaneously face multiple stresses, and it is well known, that plant responses to combined stresses are not just the merge of the different responses triggered by individual constrains (Rizhsky et al., 2004). The combination of salinity and phosphorous scarcity is very common in calcareous and alkaline soils of Mediterranean-type climate ecosystems (Zribi et al., 2012). The effect of salinity on P nutrition in crop plants is quite complex and depending among others, on the plant genotype and environmental conditions, either positive, negative or no effect of salinity on the plant P status has been reported (reviewed by Grattan and Grieve, 1999).

Arbuscular Mycorrhizal symbiosis had been defined by Parniske (2008) as “the mother of plant root endosymbiosis,” in natural ecosystems, AM symbiosis is also one the most widespread plant strategies to cope with abiotic and biotic stresses. It has traditionally been related with improved water and nutrient acquisition, especially P, However, research conducted during the last decades have drawn a more complex picture and, for example, AM have been reported to be involved in nutrient use efficiency, photosynthesis, respiration and plant metabolism (Fay et al., 1996; Del-Saz et al., 2017; Romero-Munar et al., 2017).

As components of soil-plant natural ecosystems, the symbiosis with AM fungi can ameliorate the plant’s response to salinity and have beneficial effects on plant growth and yield, which made AM fungi suitable candidates to bio-ameliorate salinized soils (reviewed by Evelin et al., 2009). The positive growth response in mycorrhized salt-stressed plants was related to an AM fungi-mediated amelioration of nutrient acquisition, especially phosphorous, when under saline conditions Pi absorption was greatly decreased (Shokri and Maadi, 2009). AM symbiosis was also reported to reduce Na^+^ uptake and translocation while favored the uptake of essential cations such as K^+^, Ca^2^*g*^+^ and Mg^2+^ and increased the K^+^/Na^+^ and Mg^2+^/Na^+^ ratios in shoots (Giri et al., 2003; Giri and Mukerji, 2004; Colla et al., 2008). However, large variation in the effectiveness of AM symbiosis to salinity and phosphorous deficiency depending on plant and AM fungi genotypes has been reported (Tian et al., 2004; Juniper and Abbott, 2006; Zou and Wu, 2011; Zribi et al., 2012).

Second-generation biofuels mainly developed in the second half of the 2000s in response to social concerns over environmental and food security issues raised by the first-generation biofuels. To ensure a more sustainable used of agricultural soils and to prevent the displacement of food crops, second-generation energy crops can be grown on marginal lands, abandoned or unsuitable for food production (Mohr and Raman, 2013). Nonetheless, marginal lands often compromised the crop establishment success due to the harsh conditions that plantlets face in these extreme environments (Pilu et al., 2012).

*Arundo donax* L. is one of the most promising species for second-generation biofuel production because its high biomass production (Hidalgo and Fernandez, 2001; Shatalov and Pereira, 2002; Lewandowski et al., 2003). Moreover, giant reed has also been reported to be an environmentally sustainable, low-cost, low-maintenance crop with very low fertilizer requirements (Lewandowski et al., 2003). However, there are some bottlenecks regarding giant reed physiology and cultivation. In nature, due to the lack of viable seeds, giant reed principally propagates through rhizomes, while plants obtained through micro-propagation of embryogenic callus are nowadays used for large-scale cultivation. This process suppresses the possibility of the mutualisms or symbiosis that happened between plantlets and soil microorganisms in natural conditions, including AM. In this line, the early inoculation of giant reed plants with AM fungi has been proposed as an useful strategy to improve field establishment and first year crop production as well as plant tolerance to marginal lands (Baraza et al., 2016; Romero-Munar et al., 2018). In view of the foregoing, we hypothesized that AM symbiosis could be a good tool to enhance *A. donax* physiological traits and biomass production in early stages under salinity and low Pi soil growing conditions, through changes in phosphorus use efficiency and sodium toxicity management. The main objective of the present work was to study the effect of AM symbiosis on the growth and biomass allocation, water relations, nutrient use efficiency and ion concentration of *A. donax* grown at different salinity regimes and phosphorous concentrations.

## Materials and Methods

### Plant and Fungi Material

Fifty-four micropropagated bare-root plants of *A. donax* K12 clone were provided by Biothek Ecologic Fuel S.L. Upon arriving, they were immediately planted in trays filled with agricultural substrate previously tindalized at 120°C for 60 min (during three consecutive days to excluding other microorganisms present in the peat) which consisted of nutrient-rich black peat (Kekkilä DSM 1 W, pH 5.9, 90% of organic matter). Principal compounds of black peat used: Sphagnum peat; additives: N-P_2_O_5_-K_2_O (16-4-17, 0.60 g l^–1^), wetting agent (0.10 g l^–1^) and dolomite limestone (5.0 g l^–1^).

One-week-old plants were transplanted in sterilized silicic sand on 4L pots. Eighteen plants were inoculated in the transplanted moment with 5 mL (5 g aprox.) of commercial inoculum (AEGIS SYM^®^), mixture of the generalist fungi *Funneliformis mosseae* (*T.H. Nicolson & Gerd.*) *C. Walker & A. Schüßler* and *Rhizophagus intraradices* (*N.C. Schenck & G.S. Sm.*) *C. Walker & A. Schüßler* (Schüßler and Walker, 2010). It was chosen as its beneficial effect on the growth of *A. donax* was previously reported (Baraza et al., 2016). Both fungi species are generalist with high richness in all soil types and commonly present in commercial inocula.

The inocula contained 25 spores per gram of each specie. Inoculated plants were termed AM plants. The rest 36 non-inoculated plants were supplied with 5 mL (5 g approx.) of autoclaved inoculum plus 3 mL of an inoculum filtrate (<20 μm) to provide a general microbial population, free of AM propagules (Bárzana et al., 2012).

Plants were grown for 3 months in a growth room under controlled conditions at 25/20°C day/night temperature, above 40% relative humidity and 12 h photoperiod (300 μmol m^–2^ s^–1^ of photosynthetic photon flux density, PPFD).

### Treatments Establishment

Pots were kept at field capacity by watering the plants with 25% modified Hoagland nutrient solution with 2.5 μM Pi and 1 mM Na for 7 weeks to allow AM fungi establishment. After AM colonization, two phosphorus and three salinity treatments where set up in a step-wise manner in sextuplicate, resulting in nine treatments. Nutritional factor (N): Control, C (non-inoculated plants growing with 2.5 μM P); Phosphorus plants, P (non-inoculated plants growing with 0.5 mM Pi); and arbuscular mycorrhiza plants, AM (colonized plants growing with 2.5 μM Pi); each N treatment was combined with three salt concentrations (S): 1, 75 and 150 mM NaCl. Before, N and S treatments began, the stem length (cm) was measured to ensure the homogeneity in size among plantlets (C, P and AM, 82.57 ± 2.43, 84.88 ± 2.38 and 82.78 ± 2.65, respectively, *p* = 0.76).

### Mycorrhizal Colonization

The percentage of mycorrhizal root colonization was determined at the end of the experiment, 2 months after starting treatments (3-month after inoculation). It was assessed by visual observation of fungal colonization. Roots were digested with 10% KOH and stained with trypan blue (0.05% in lactic acid (v/v), according to Phillips and Hayman (1970). AM colonization was assessed using the magnified intersections method (Abbott and Robson, 1984), where the frequency of colonization represents the ratio between fragments of colonized root and the total number of root fragments examined. An average of 300 root pieces per plant and six plants per treatment were examined. Percentage of mycelium, spores, vesicles, arbuscules and total inoculation were determined from roots to evaluate salinity effect on the average of these structures.

### Biomass Measurements

Two months after starting treatments, total number of leaves, stems and length of the highest stem were measured in six plants per treatment.

To assess the biomass of the different plant tissues, each plant was divided into leaves (grouped in: young leaves, YL – two leaves from the top of the stem; mature leaves, ML – third or fourth leaf from the top of the stem; and old leaves, OL – leaves located at the bottom of the stem), stems and roots (grouped in thick roots, TR – diameter >5 mm; and fine roots, FR – diameter <5 mm). To measure the dry weight (DW), plant tissue was dried in a forced-air oven at 70°C for 72 h. The roots were washed in distilled water before drying.

The mycorrhizal dependency (MD) was calculated for each treatment according to Plenchette et al. (1983): MD (%) = (DW of mycorrhizal plant/DW of non-mycorrhizal plant)/DW of mycorrhizal plant × 100. While the mycorrhizal growth response was calculated according to Hoeksema et al. (2010): MGR = log_e_ [DW of mycorrhizal plant/DW of non-mycorrhizal plant].

Specific leaf area (SLA) was determined as the one-sided area of the fourth leaf of each plant divided by its oven-dry mass.

Whole-plant leaf area (LA) was calculated as the total leaf dry weight/SLA.

### Leaf Physiology

Physiological parameters were measured in the leaves, 2-month after treatment establishment.

Gas exchange parameters were measured from 10:00 to 12:30 h on one ML per plant leaves using an open infrared gas exchange analyzer system (Li- 6400; Li-Cor Inc., Lincoln, NE, United States). Leaf chamber fluorometer (Li-6400-40, Li-Cor Inc.) conditions were PPFD of 1.500 μmol m^–2^ s^–1^, with 10% of blue light, and a vapor pressure deficit of 2.0–3.0 kPa at a CO_2_ concentration of 400 μmol mol^–1^(air). The leaf temperature was set at 25°C, and the relative humidity of the incoming air was approximately 50% throughout all measurements.

To quantify chlorophyll and leaf protein concentrations, samples of the leaves used for gas exchange measurement (ML) were frozen in liquid nitrogen and stored at −80°C. Photosynthetic pigments were extracted using 96% ethanol. Chlorophyll a, chlorophyll b and total chlorophyll content were calculated according to Lichtenthaler and Wellburn (1983). Leaf protein content was determined following the method described by Bradford (1976).

Leaf osmotic potential was measured in two mature leaves per plant. Frozen samples of ML were thawed and grinded for 30 s. A sap volume of 10 μL was used (Gucci et al., 1991) to determine the leaf osmotic potential using a Wescor 5500 vapor pressure osmometer (Wescor Inc., Logan, UT, United States).

### Ion Tissue Concentration

Ion concentration was determined in YL, ML, OL, S, TR, and FR of each plant. Dry tissue was finely powdered using an orbital shaker in seal tubes with glass balls. One hundred mg of dried tissue were ashed at 550°C. After cooling, the ash was washed into polyethylene tubes with 9.2 ml 0.08 M H_2_SO_4_ and 0.8 ml 40% HF were added. The suspension was shaken for 1 h and left overnight at room temperature. One micro liter of the suspension was transferred into polyethylene tubes with 25 ml of 0.32% H_3_BO_3_ to neutralize the excess of HF prior analysis (Xia et al., 2000). After filtering, the Na^+^, K^+^, Ca^2+^, Mg^2+^, Pi and Si concentrations were determined by Inductively Coupled Plasma (ICP) Spectrometry (Perkin-Elmer Plasma-2000, Perkin-Elmer Inc., Norwalk, CA, United States).

The nutrient use efficiency was calculated dividing the total plant biomass by the plant nutrient concentration (Siddiqi and Glass, 1981).

External and internal phosphorus use efficiency (PUE), PUE_e_ and PUE_i_, respectively, for leaf, stem and root tissues were calculated as:

PUE_e_ = Tissue DW/plant P concentration, according to Zhang et al. (2009)
PUE_i_ = Tissue DW/tissue P concentration, following Hammond et al. (2009)

### Statistical Analyses

All datasets were tested for a normal distribution and variance homogeneity (*P*<0.05), and variables were log transformed when necessary. Two-way analysis of variance (ANOVA II) was performed to analyze the effect of the two factors (N and S) in the main studied parameters: biomass dry weight, leaf physiological parameters and ion tissue concentration. We performed a *post hoc* Tukey test to analyze differences among the nine treatments.

The analyses were performed using the JMP^®^, Version 10 (SAS Institute Inc., Cary, NC, United States 1989–2007).

## Results

### Salinity Severely Decreased AM Symbiosis Colonization

**FIGURE 1:**
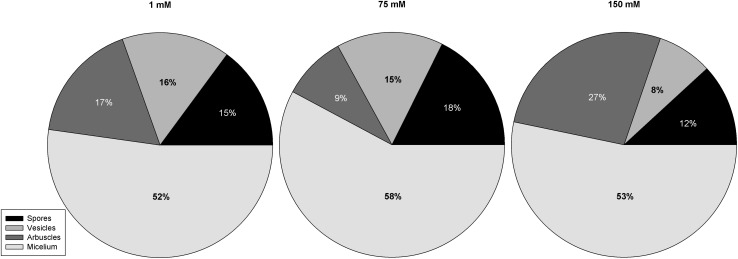
AM fungi structures percentages to the total colonization of each salinity treatment.

The colonization of *A. donax* roots by *R. intraradices* and *F. mosseae* reached values of 54% ± 9.98 in non-salinized plants. Despite root colonization was greatly reduced by S factor (*p* = 0.0196, ANOVA), but differences between three salt treatment were partially significant with values of 23.50% ± 6.22 and 12.29% ± 4.33 in 75 and 150 mM NaCl-treated plants, respectively. Tukey test showed significant differences between 1 and 150 Mm, but not between 1 and 75 mM neither between 75 and 150 mM. Salinity also significatively reduced the presence of spores (*p* = 0.0325, ANOVA), vesicles (*p* = 0.0445, ANOVA) and arbuscules (*p* = 0.0435, ANOVA) observed in roots. As well as in total colonization, *post hoc* Tukey test showed significant differences between 1 and 150 NaCl mM treated plants, but not between them and 75 mM NaCl, in percentages of spores and vesicles. The amount of arbuscules was highly reduced (47%) at moderate salt stress (75 mM NaCl), while it greatly increased (59%) at severe salt stress (150 mM NaCl) with respect non-salinized roots, but those differences were not significant according to Tukey test (Figure 1). C and P plants were also screened and no colonization was detected.

### AM Symbiosis Increased Plant Growth in Non-salinized and Moderately Salinized Plants Under Low P Condition

**FIGURE 2:**
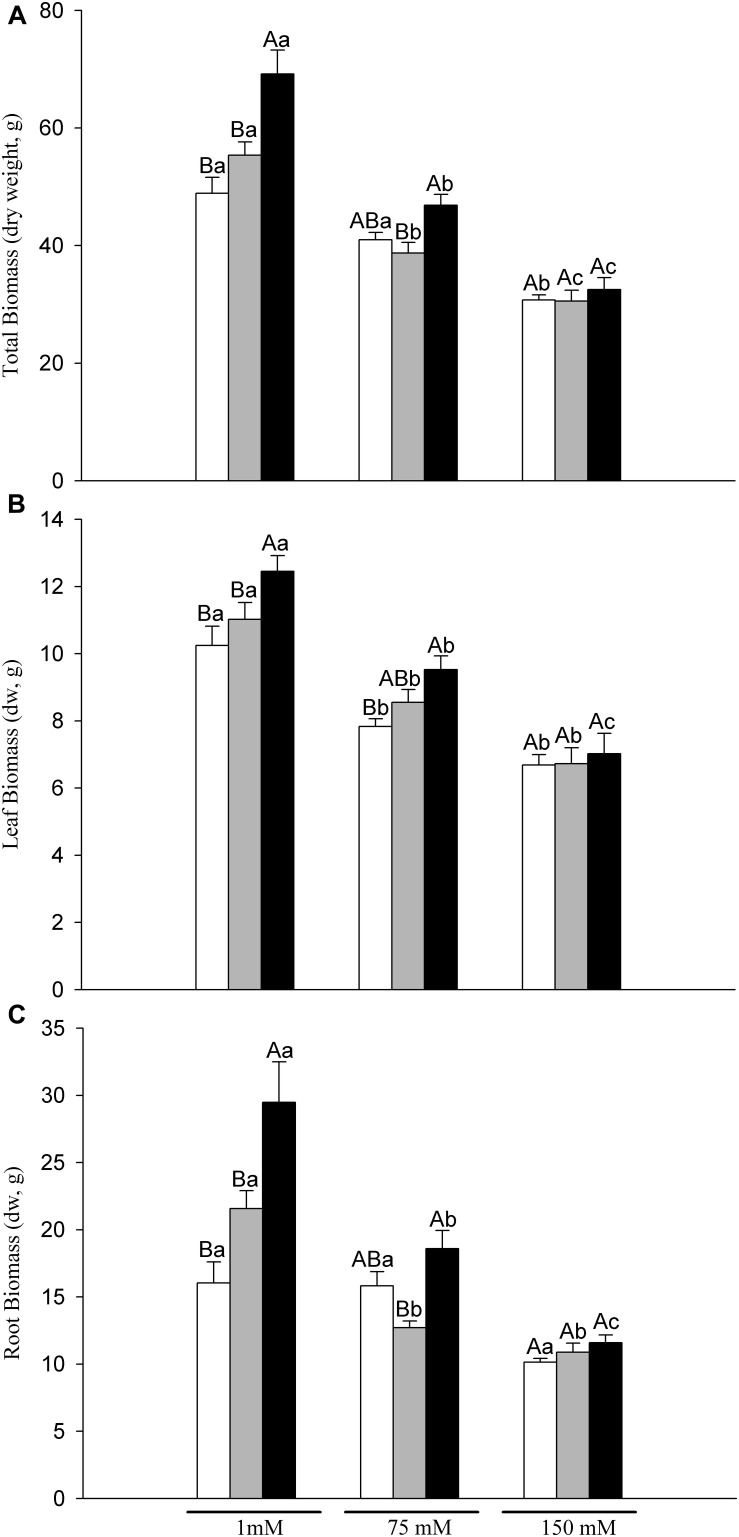
Total biomass **(A)**, leaf **(B)** and root **(C)** dry weight of control (C, white bar), phosphorus (P, gray bar) and arbuscular mycorrhiza plants (AM, black bar) grown at 1, 75 and 150 mM NaCl. Values are mean ± SE of six replicates. Different capital letters indicate significant differences among nutritional levels (C, P and AM) within the same salt level, and lowercase letters indicate significant differences among salt levels within the same nutritional level, from *post hoc* Tukey test.

Total plant biomass and its distribution were significantly affected by the nutritional and salinity treatments (N and S factor, respectively), were AM plants showed the highest value, and total biomass decreased from 1 mM >75 mM >150 mM. The interaction of the two factors was also significant (Table 1A and Figure 2A).

**TABLE 1A:** Summary of two-way analysis of variance (ANOVA) and *R* saquere adjusted of the total model (*R*^2^ adj) for the effects of nutrient treatment (N) and salt treatment (S), with their interaction factor (N × S) on biomass, physiological and nutrition- related traits in *Arundo donax* plants.

**Trait**	***R^2^ adj***	***N***	***S***	***N* × *S***
*Total Biomass*	0.86	**<0.0001**	**<0.0001**	**0.0007**
Leaf Biomass	0.81	**<0.0001**	**<0.0001**	0.0992
*Root Biomass*	0.76	**<0.0001**	**<0.0001**	**0.0005**
*LA (cm^2^)*	0.51	**0.0053**	**<0.0001**	0.5157
*A*_N_	0.56	0.1174	0.1656	**0.0007**
*g*_s_	0.57	0.0704	0.1736	**<0.0001**
*A*_N_/*g*_s_	0.57	0.0354	0.8879	**0.0007**
Ψπ	0.30	0.5834	**<0.0001**	0.2783
Leaf protein	0.30	0.3107	0.8212	**0.0009**
Chl a	0.25	0.2869	0.0576	**0.0080**
Na/K (leaf)	0.55	0.1221	**<0.0001**	**0.0297**
P_i_	0.69	**<0.0001**	0.3232	**0.0165**
Mg^2+^	0.07	0.4631	0.0723	0.5191
Ca^2+^	0.03	0.2670	**0.0489**	0.8620
Si	0.03	0.0715	0.3612	0.9733
KUE	0.64	**0.0017**	**<0.0001**	**0.0471**
MgUE	0.51	**0.0072**	**<0.0001**	0.2900
CaUE	0.22	0.1207	0.9261	0.7680
SiUE	0.32	0.7572	**0.0045**	0.9357
PUE	0.61	**<0.0001**	**<0.0001**	0.5626
PUE_e_ Leaf	0.68	**<0.0001**	**<0.0001**	0.1906
PUE_e_ Stem	0.62	**<0.0001**	**<0.0001**	0.2791
PUE_e_ Root	0.71	**<0.0001**	**0.0004**	0.1323
PUE_i_ Leaf	0.69	**<0.0001**	**<0.0001**	0.1985
PUE_i_ Stem	0.57	**<0.0001**	**<0.0001**	1.7833
PUE_i_ Root	0.88	**<0.0001**	**<0.0001**	**0.0108**

*Values correspond to *P* values. Abbreviations as shown in the text. Significant *P* values are highlighted in bold.*

**TABLE 1B:** Summary of three-way analysis of variance (ANOVA) and R saquere adjusted of the total model (R^2^ adj) for the effects of nutrient treatment (N), salt treatment (S) and tissue (T) with their interaction (N × S, T × N, T × S and T × N × S) on sodium (Na^+^, mM) and potassium concentration (K^+^, mM).

	***R*^2^*adj***	***N***	***S***	***N* × *S***	***T***	***T* × *N***	***T* × *S***	***T* × *N* × *S***
Na^+^	0.91	**<0.0001**	**<0.0001**	**0.0044**	**<0.0001**	**0.0080**	**<0.0001**	**<0.0001**
K^+^	0.92	0.4362	**<0.0001**	0.6401	**<0.0001**	0.1936	**<0.0001**	0.8449

*Values correspond to *P* values. Significant *P* values are highlighted in bold.*

In non-salinized plants, P and AM treatments increased total plant dry weight by 11 and 41%, respectively with respect to C plants, which was mainly due to higher root rather than higher leaf biomass (Figures 2B,C). This significant increase in AM plants was linked with 30% of mycorrhizal dependency (MD) observed (Table 2). Under mild salinity (75 mM NaCl), no significant differences in total biomass between P and C treatments were observed. However, regardless the important decrease in AM root colonization and MD caused by salt (Table 2), AM plants showed a 14% increase in total dry biomass due to similar positive AM effects on leaf and root growth. In 150 mM NaCl-treated plants, no differences on total, leaf or root biomass between nutritional treatments were found (Figures 2A–C, respectively).

**TABLE 2:** Mycorrhizal dependency (MD) and mycorrhizal growth response (MGR) of A. donax under three levels of NaCl concentration.

**NaCl (mM)**	**MD (%)**	**MGR**
1	29.33	0.35
75	12.56	0.13
150	5.35	0.06

*It was calculated respect C plants.*

Regarding biomass distribution (Table 3), at 1 mM NaCl, AM and P plants showed higher root-to-shoot ratio than C plants while under mild salt stress, no significant differences among nutritional treatments were observed.

**TABLE 3:** Biomass parameters of C, P and AM plants grown at 1, 75 and 150 mM NaCl.

**S factor**	**1**			**75**			**150**		
**N factor**	**C**	**P**	**AM**	**C**	**P**	**AM**	**C**	**P**	**AM**
LA (cm^2^)	543±40^b^	574.5±51^b^	755±120^a^	403±23^a^	421±26^a^	439±20^a^	351±40^a^	341±24^a^	400±37^a^
S:R	0.49±0.05^c^	0.64±0.03^ab^	0.74±0.06^a^	0.63±0.04^b^	0.49±0.02^a^	0.66±0.06^a^	0.50±0.02^a^	0.56±0.02^a^	0.57±0.03^a^
SLA (cm^2^ g^–1^)	53±3^a^	51.7±3^a^	51.8±4^a^	51.5±2^a^	49.3±2.6^a^	46.2±1.5^a^	51.8±4^a^	51±2^a^	57±2.7^a^
LAR (cm^2^ g^–1^)	11±0.5^a^	10.4±0.9^a^	9.5±1.2^a^	9.9±0.7^a^	11±0.9^a^	9.4±0.4^a^	11.4±1.2^a^	11.2±0.6^a^	12.3±0.9^a^
LMR (g g^–1^)	0.211±0.01^a^	0.199±0.01^a^	0.183±0.01^a^	0.191±0.01^a^	0.222±0.01^a^	0.203±0.01^a^	0.217±0.01^a^	0.219±0.004^a^	0.215±0.01^a^

*Total leaf area [LA, (cm^2^)], root to shoot ratio (R:S) Specific leaf area [SLA, (cm^2^ g^–1^)]; leaf area ratio [LAR, (cm^2^ g^–1^)] and leaf mass ratio [LMR, (g g^–1^)]. Data are means ± SE of 5 to 6 replicates. Different letters indicate statisticaly significant differences among N treatments with the same S factor (*p*-values are shown in Table 1).*

Nutritional and salinity factors had an independent effect on leaf area (Table 1A). In non-salinized plants, P treatment did not increase leaf area, however, AM plants showed statistically higher leaf area than P and C plants (Table 3). Non-significant differences among nutritional treatments were observed neither at 75 mM NaCl nor at 150 mM NaCl (Table 3). Furthermore, no differences in SLA, leaf area ratio (LAR) and leaf mass ratio (LMR) among nutritional or salinity treatments were found (Table 3).

### Physiological *Arundo* Leaf Responses to Different Nutritional and NaCl Treatments

**FIGURE 3:**
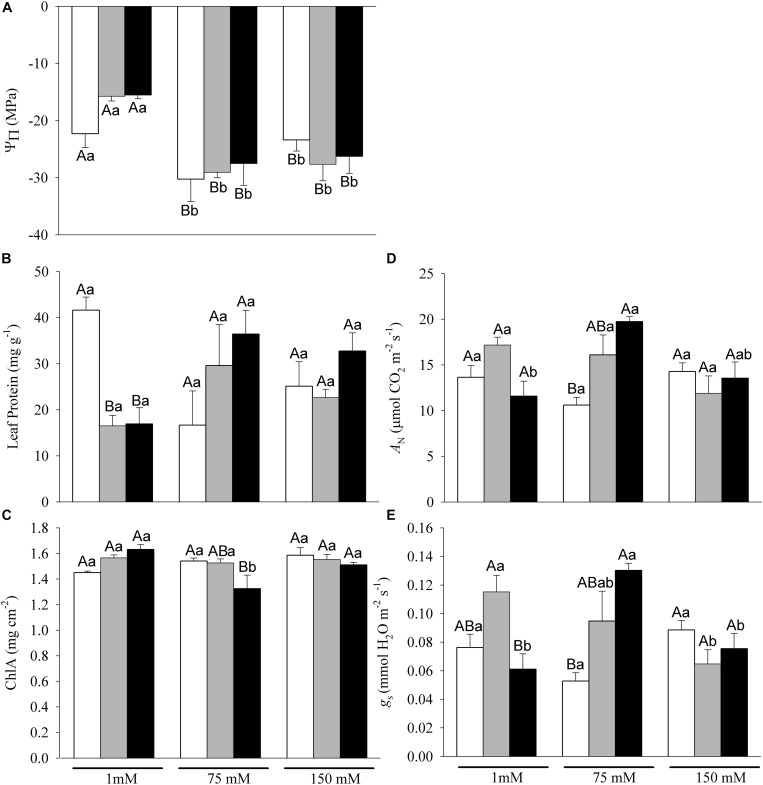
Leaf physiological traits: **(A)** Osmotic potential (Ψπ), **(B)** Leaf protein, **(C)** Chlorophyll a concentration (Chl a), **(D)** Net Photosynthesis rate (*A*_N_), **(E)** Stomatal conductace (*g*_s_) of C, P and AM plants (white, gray and black bars, respectively) at 1, 75 and 150 mM NaCl. Values are mean and SE of six replicates. Different capital letters indicate significant differences among nutritional levels (C, P and AM) within the same salt level, and lowercase letters indicate significant differences among salt levels within the same nutritional level, from post hoc Tukey test.

Leaf physiological responses of C, P and AM plants grown at 1, 75 or 150 mM NaCl are shown in Figure 3. Under non-salt stress, C plants showed statistically significant lower osmotic potential than P and AM plants, while under salt stress conditions, osmotic potential declined with increased salinity with no differences among nutritional treatments (Figure 3A).

Regarding leaf protein concentration (Figure 3B), only the interaction between nutritional and salinity (N × S) was significant (Table 1A). According to *post hoc* Tukey test, P plants showed significantly lower leaf protein content than C plants under non-salt stress conditions. No differences were observed in leaf protein among N levels at 75 and 150 mM.

Salinity and N factor did not affect chlorophyll a separately, although significant N × S interaction was found (Table 1A). AM showed higher chlorophyll a concentration under non-salt stress conditions, than C plants (Figure 3C). Moderate salt stress did not affect chlorophyll a in C and P plants, whilst in AM plants, chlorophyll a decreased significantly and showed the lowest concentration (*post hoc* Tukey). No significant differences in chlorophyll a were found among nutritional treatments at 150 mM NaCl.

Photosynthesis (*A*_N_) and stomatal conductance (*g*_s_) (Figures 3D,E, respectively) were significantly affected by nutritional and salinity interaction (N × S, Table 1). Under non-saline conditions, P plants showed significantly higher *A*_N_ and *g*_s_ than plants grown under low P. However, under mild salt stress conditions, AM plants significantly increased both parameters respect to C and P plants. No differences were found at 150 mM NaCl among N levels.

### AM Symbiosis Modulated Salinity and Low Phosphorus Effects on Ion Uptake and Distribution

**FIGURE 4:**
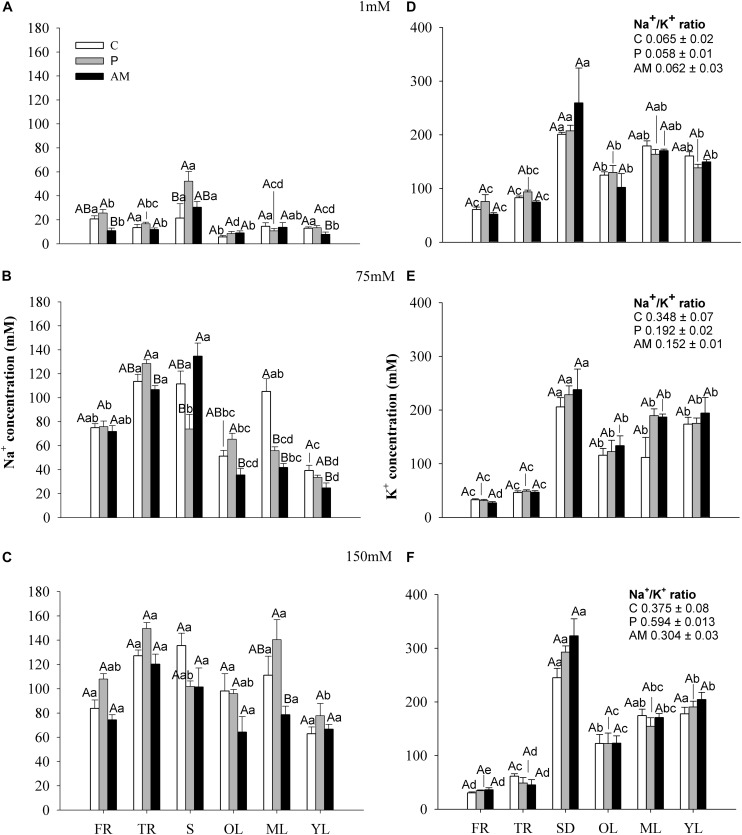
Sodium and potassium concentration: **(A–C)** show Na^+^ concentration (mM), and **(D–F)** show K+ concentration (mM) of different tissues (fine root – FR; thick root – TR; stem – S; old leaf – OL; mature leaf – ML and young leaf – YL) at 1, 75 and 150mM salt levels, of C, P and AM plants (white, gray and black bars, respectively). Values of ML Na^+^/L^+^ ratio are show in a nested chart. Values are mean and SE of six replicates. Different capital letters indicate significant differences among nutritional levels (C, P and AM) within the same tissue, and lowercase letters indicate significant differences among tissues within the same nutritional level, from post hoc Tukey test.

Nutritional and salinity factors had a dependent effect on tissue Na^+^ concentrations (Table 1B). Furthermore, the Na^+^ concentration profile among the different tissues was dependent on the nutritional treatment (Figures 4A–C). While in non-salt stressed plants (Figure 4A), where Na^+^ was preferentially accumulated in stems, P plants showing the highest values, at 75 and 150 mM AM plants showed the lowest ones. Under mild and severe stress salt conditions, thick roots (TR) and stems (S) had the highest Na^+^ followed by fine roots (R), mature leaves (ML) and old leaves (OL) with the lowest concentrations found in young leaves (YL) (Figures 4A–C). At 75 mM NaCl, AM plants strongly reduced Na^+^ concentration in leaves, whilst C and P plants only showed significant reduction in young leaves (Figure 4B). At 150 mM, despite AM plants showed an equal Na^+^ distribution among tissues, a significant reduction of Na^+^ concentration in mature leaves respect C and P plants was observed (Figure 4C).

Potassium concentration was significantly affected by the S factor and tissue distribution and their interaction, but not by the N factor (Table 1B). The K^+^ distribution profile among the different tissues was the same in all nutritional treatments with highest values found in the aboveground tissues, especially in stems. Salinity decreased root K^+^ concentrations but its concentration was maintained in the aboveground tissues (Figures 4D–F).

The Na^+^/K^+^ ratio of ML increased in the order C > P > AM (*p* < 0.05, ANOVA and *post hoc* Tukey test) at 75 mM NaCl, while increased in the order P > C > AM (*p* < 0.05, ANOVA and *post hoc* Tukey test) at 150 mM NaCl (Figures 4D–F).

The N factor highly affected phosphorus concentration but its effect depended on the salt treatment (in Supplementary Table S1A). Significantly higher P concentration was observed in P plants, and slightly but not significantly decreases through salinity treatments. *Post hoc* Tukey test showed higher P concentration in AM plants compared with C plants under 1 mM NaCl treatment, but those differences disappeared under salinity conditions. Salt stress did not significantly affect tissue Pi concentration at any nutritional treatment (Table 1A). The Pi concentration response profile of the different plant tissues showed lower values in stems and thick roots respect to fine roots and all other aboveground tissues (in Supplementary Table S1A).

Calcium concentration was dependent on the salinity treatment while no significant differences were found in Mg^2+^ or Si concentrations among N or S treatments (Table 1A). Ca^2+^ concentrations decreased in roots and increased in leaves of P and AM plants with increasing salinity, while no differences were found among salt treatments in C plants. Calcium and Mg^2+^ showed the highest concentrations in OL, while Si was preferentially accumulated in roots and OL (in Supplementary Tables S1B–D, respectively).

### AM Symbiosis Increased Nutrient Use Efficiency

**FIGURE 5:**
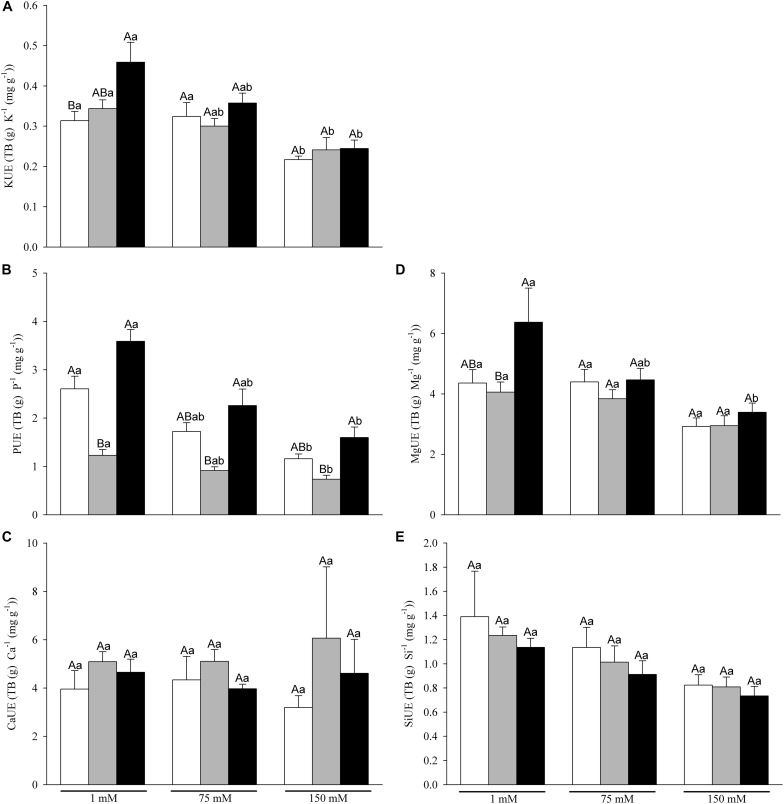
Nutrient Use Efficiency [Total Biomass, TB (g)/nutrient concentration (mg g^–1^)]. **(A)** K^+^, KUE; **(B)** P, PUE; **(C)** Mg^2+^, MgUE; **(D)** Ca^2+^, CaUE and **(E)** Si, SiUE. Values are mean and SE of six replicates of of C, P and AM plants (white, gray and black bars, respectively) at the three salinity levels (1, 75 and 150 mM NaCl). Different capital letters indicate significant differences among nutritional levels (C, P and AM) within the same salt level, and lowercase letters indicate significant differences among salt levels within the same nutritional level, from post hoc Tukey test.

Potassium use efficiency (KUE) was significantly affected by N and S factors and their interaction (Figure 5A and Table 1A). AM plants, followed by P plants, showed higher values compared to C ones at 1 mM (Figure 5A), while no differences were observed under moderate and severe stress conditions. With the increase of Na^+^ concentration, KUE was progressively decreased in all N levels.

Statistical analysis showed Mg^2+^ use efficiency (MgUE) was affected by S and N factors but not for their interaction (Table 1A). At 1 mM NaCl, AM plants showed the highest MgUE (*post hoc* Tukey test), but not differences were found among C, P and AM plants under salinity stress conditions. Ca^2+^ use efficiency (CaUE) and silicon use efficiency (SiUE) showed no statistical differences among treatments, and any statistical effect of N and S factor and their interaction (Table 1A and Figures 5C,E, respectively).

C and AM non-salinized plants showed highest phosphor use efficiency (PUE) than P plants. Regarding salinity stress conditions, the *post hoc* Tukey showed that AM plants presented higher PUE followed by C and P plants (Figure 5B) at 75 and 150 mM NaCl. However, in all N levels, salinity had a negative effect on PUE, decreasing at 75 mM and ranged the lowest values at 150 mM NaCl.

**FIGURE 6:**
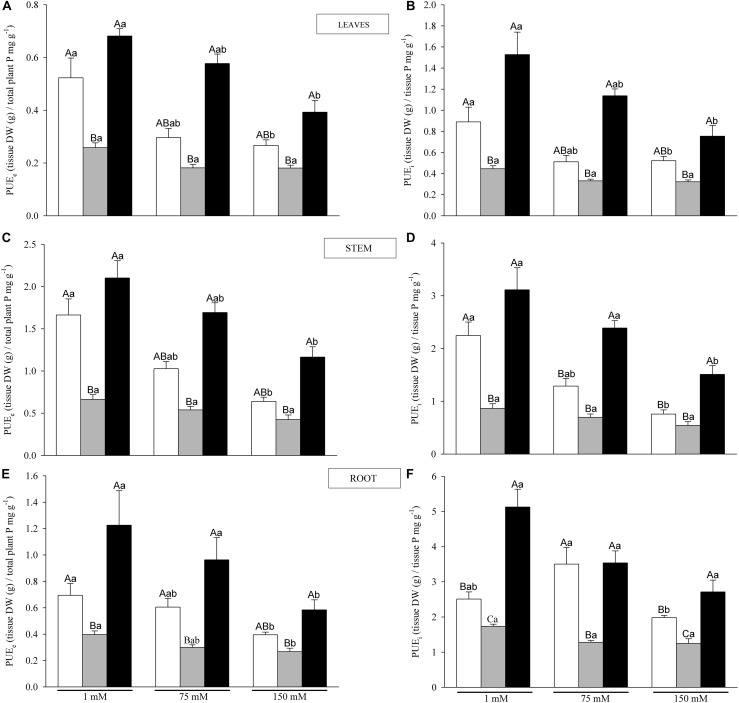
Phosphorus use efficiency external and internal (PUE_e_ and PUE_i_, respectively), of leaves **(A,B)**, shoots **(C,D)** and roots **(E,F)**, for C, P and AM plants (white, gray and black bars, respectively) under 1, 75 and 150 mM NaCl. Values are mean and standard error of at least four replicates per treatment. Different capital letters indicate significant differences among nutritional levels (C, P and AM) within the same salt level, and lowercase letters indicate significant differences among salt levels within the same nutritional level, from *post hoc* Tukey test.

Phosphorus use efficiency external and internal (PUEe and PUEi, respectively) statistical analyses showed that both were significantly affected by N and S factors and their interaction (Table 1A and Figures 6A–F). Increased salinity declined PUE_e_ (Figures 6A,C,E) and PUE_i_ (Figures 6B,D,F) in C and AM plants with little effect in P plants. PUE_e_ showed lower values than PUE_i_ in all nutritional and salinity treatments. In leaves and stems, at 75 and 150 mM NaCl, AM plants showed the highest values of PUEe and PUEi, followed by C and P plants (Figures 6A–D). The highest PUE_i_ values were found in roots, where in non-salinized conditions AM plants showed 2-fold higher values than C plants (Figure 6F).

## Discussion

In arid and semiarid regions with calcareous soils, combined phosphorus deficiency and high salinity are often soil-borne stress conditions that limit crop establishment and plant production (Bargaz et al., 2018). Although unsuitable for food crops, such marginal lands have potential for growing low-cost, environmentally sustainable, energy crops which could also enhance ecosystem services such as carbon sequestration and soil structure. *A. donax* is a fast-growing perennial grass which has been highlighted as a promising crop for lignocellulosic biomass production in salinized soils (Sánchez et al., 2016 and references herewith). On the other hand, AM symbiosis is known to ameliorate the plant response to constraining factors in calcareous-salinized soils by enhancing Pi acquisition (Harrison and van Buuren, 1995; Wright and Upadhyaya, 1998; Richardson et al., 2009; Smith and Smith, 2011) and salinity tolerance (Mohammad et al., 2003; Evelin and Kapoor, 2014).

Here, marginal land stress conditions were mimicked by growing AM and non-AM *A. donax* plants at low and sufficient phosphorous concentrations under increasing salinity. To the best of our knowledge, few studies have focused on the role of the AM symbiosis in the plant responses to combined salt stress and low P (Mohammad et al., 2003; Del-Saz et al., 2017).

The effect of concurrent abiotic stresses on plant growth is related to both, the severity of each individual stress and the plant species (Rabhi et al., 2007; Slama et al., 2008) with plant growth being determined by the most growth-limiting stress factor (van der Ploeg and Kirkham, 1999; Talbi Zribi et al., 2011).

Regardless the marked differences between C and P plants in total Pi tissue, whose values were in the high range in P plants (>5 mg P/g DW) and close or just below the critical values (3 mg P/g DW) in C plants (Veneklaas et al., 2012 and references herewith), the low Pi concentration supplied in this study did not trigger Pi scarcity-related growth responses in *A. donax*. Non-salinized C plants did not show increased root/shoot biomass ratio or decreased chlorophyll production (Hammond and White, 2011), neither a decrease in CO_2_ assimilation. Cytokinins (CKs) have been related to Pi signaling, with low-P conditions decreasing plant CK concentrations (Rouached et al., 2010). The maintenance of CK signaling could at least partly explain why plant growth, shoot/root ratio and leaf chlorophyll in C and P plants were alike. While similar photosynthesis values in C and P plants could be explained by the highest external and internal PUE ratio in C compared with P plants since internal PUE at leaf level is a key factor to prevent a reduction in photosynthesis due to increased sucrose concentrations in response to low P (Yang et al., 2017).

The symbiosis of *A. donax* with AM fungi (AM plants), showed the best ameliorating growth response to low P. Root and leaf biomass in AM plants even surpassed the P plants values. AM symbiosis has frequently been reported as a biological method to promote plant growth by increasing nutrient uptake, especially P (Wright and Upadhyaya, 1998; Richardson et al., 2009; Smith and Smith, 2011). However, variation in the response from positive to negative to AM symbiosis exits depending on the plant and fungus genotype and the environmental/agronomic conditions (Dai et al., 2014; Johnson et al., 2015). There are several works reporting the positive effects of AM symbiosis in *A. donax* (Tauler and Baraza, 2015; Baraza et al., 2016; Romero-Munar et al., 2017, 2018), but also negative or null effects (Pollastri et al., 2018), depending on growing conditions, fungi partners and/or the phenological stage of the plants (Johnson et al., 1997; Smith and Smith, 2015).

Despite the high resilience shown by *A. donax* to low P conditions, AM symbiosis provided this species with additional benefits as a 30% degree of plant growth change was associated with AM colonization.

However, in contrast with previous studies (Wright and Upadhyaya, 1998; Richardson et al., 2009; Smith and Smith, 2011), the increased growth response in AM plants was related to higher nutrient use efficiency rather than to tissue P concentration. Higher KUE, PUE and MgUE could have benefited, amongst others, leaf water relations and photosynthesis and consequently plant growth. Moreover, the greatest internal root PUE in AM plants could be due to an inhibitory effect of the AM-inducible root P transporters pathway on the direct uptake pathway (Smith et al., 2004; Campos-Soriano et al., 2012), and the activation of the mycorrhizal nutrient uptake pathway, an energy saving mechanisms (Watts-Williams et al., 2015). In roots of plants grown under P limitation, AM colonization decreases both carboxylates exudation and respiration, and enhances biomass production (Del-Saz et al., 2017).

Regarding to the effect of concurrent abiotic stresses on plant growth above mentioned, in the present work, the most growth-limiting stress factor was salinity, since both, as single stress (P plants) or in combination with low phosphorus (C and AM plants), moderate and severe salt stress conditions greatly reduce plant biomass. Despite some studies have listed *A. donax* as a salt tolerant species (Williams et al., 2008; Sánchez et al., 2015), our results showed a fast inhibition of leaf growth in this species caused by salt independently of P supply, which compromised carbon acquisition and consequently yield, due to its dependence on leaf production and expansion. However, AM symbiosis ameliorated the response of *A. donax* to combine low P and mild salinization conditions. At 75 mM NaCl, when C and P plants growth was driven by salinity rather than P availability, AM plants showed 14% higher biomass than C and P, despite the important reduction of AM root colonization and consequently in MD. Although AM symbiosis are present in saline soils (Landwehr et al., 2002), the osmotic and toxic effects of salt not only affect the host plants but also the fungi in a similar way (Juniper and Abbott, 2006). In fact, the effect of salinity on plant colonization by AM depends on the fungus tolerance to salinity (Yamato et al., 2008). Under our experimental conditions, root colonization by *R. intraradices* and *F. mosseae* was severely decreased by salt. Reduced root colonization by AM fungi in saline environments has been related to a salt effect on primary infection as more inhibition has been reported at the early stages of AM symbiosis (Wilson, 1984; McMillen et al., 1998). However, in this study, salinity treatments were started after AM colonization was achieved and therefore the reduction in AM colonization was more likely due to a salt effect on secondary colonization. On the other hand, the percentage of root colonization is not directly related to the symbiotic outcome (Giri and Mukerji, 2004). In this line, at 75 and 150 mM NaCl, AM plants showed remarkable growth and sodium management responses compared with C and P plants.

Sodium specific toxic effects have been associated with the built-up of high leaf Na^+^ concentrations (Munns, 2002). In barley, a salt-tolerant species, plant dry weight was found to decrease at shoot Na^+^ concentrations above 9.2 mg g^–1^ DW (Tavakkoli et al., 2011). Notably, similar and even higher Na^+^ values were found in leaves of C and P plants submitted at the highest salt treatment, while leaf Na^+^ was significantly lower in AM plants, pointing out to an enhanced effect of AM fungi on the Na^+^ exclusion capacity of *A. donax.* It has been reported that AM symbiosis increased and also orchestrated Na^+^ exclusion response (Giri and Mukerji, 2004). In AM plants the intraradical hyphae could have provided the plant with an additional space for Na^+^ allocation and help to prevent its translocation to the shoots (Cantrell and Linderman, 2001). This higher Na^+^ exclusion capacity could be related to the ameliorated growth found in AM plants grown at mild salt conditions. Furthermore, it has been propose that AM fungi excludes Na^+^ by discrimination in its uptake from the soil or during its transfer to plants (Hammer et al., 2011), but also had specific and high affinity phosphorus transporters (Harrison et al., 2002), could explain how AM plants maintain the internal and external PUE and reducing Na^+^ concentration in tissues, under moderate and severe salinity and low P availability, compared to C but also than P plants.

To summarize, the results indicate that AM symbiosis could be a good tool to enhance *A. donax* physiological traits and biomass production under combined low phosphorus and salt stress conditions during the plant’s early developmental stages. Despite the negative impact of high salt on AM colonization, AM plants were able to maintain cellular homeostasis at low Pi supply by assessing higher PUE rather than increasing tissue P concentrations. Thus, AM symbiosis establishment at early development stages could play a key role in the *A. donax* cultivation in marginal lands.

## Author Contributions

AR-M and CC designed and performed the research, collected, analyzed and interpreted the data, and wrote the manuscript. JG and EB collaborated on data interpretation and writing the manuscript.

## Conflict of Interest Statement

The authors declare that the research was conducted in the absence of any commercial or financial relationships that could be construed as a potential conflict of interest.
