# Supplementary material for: Arbuscular Mycorrhizal Fungi Confer Salt Tolerance in Giant Reed (Arundo donax L.) Plants Grown Under Low Phosphorus by Reducing Leaf Na+ Concentration and Improving Phosphorus Use Efficiency
Source: Front Plant Sci. 2019 Jul 16;10:843. doi: 10.3389/fpls.2019.00843 (PMC6664208; doi:10.3389/fpls.2019.00843)
Supplement: Supplementary file 1 [file Table_1.pdf]

Supplementary Information (SI)

Ion concentration and distribution

Supplementary Table S1. Ion concentration (mg g^{-1}) obtained in each tissue analyzed by ICP, were SU = stem up (distal part of the stem); YL = young leaf; ML = mature leaf (fully expanded leaf); OL = old leaf (leaf in senescence); SD = stem down (proximal part of the stem); TR = thick root and FR = fine root. Values are mean ± SE of at least four replicates per treatment.

A. Phosphorus plant distribution

P mg g^{-1}	1			75			150		
Tissue	C	P	AM	C	P	AM	C	P	AM
SU	2.29 ± 0.18	6.31 ± 0.35	2.12 ± 0.65	4.85 ± 0.81	7.51 ± 0.79	4.81 ± 1.21	5.92 ± 0.67	7.33 ± 0.41	4.40 ± 0.47
YL	4.23 ± 0.51	7.69 ± 0.35	4.03 ± 0.39	5.67 ± 0.64	7.90 ± 0.65	5.34 ± 0.68	7.30 ± 1.57	7.30 ± 0.37	4.93 ± 0.49
ML	2.73 ± 0.40	7.95 ± 0.44	2.53 ± 0.18	4.15 ± 0.06	7.20 ± 0.44	3.06 ± 0.30	4.37 ± 0.33	10.5 ± 0.74	3.60 ± 0.17
OL	2.94 ± 0.50	9.01 ± 1.00	2.67 ± 0.23	3.01 ± 0.60	7.53 ± 0.94	3.55 ± 0.49	3.41 ± 0.40	6.62 ± 1.25	3.30 ± 0.53
SD	0.55 ± 0.05	1.37 ± 0.13	1.02 ± 0.38	0.72 ± 0.05	2.91 ± 0.51	0.95 ± 0.14	1.63 ± 0.34	2.09 ± 0.30	1.26 ± 0.17
TR	1.88 ± 0.20	4.48 ± 0.23	2.42 ± 0.08	1.61 ± 0.08	3.69 ± 0.12	2.33 ± 0.30	2.21 ± 0.32	3.53 ± 0.14	1.97 ± 0.06
FR	2.88 ± 0.28	6.18 ± 0.50	3.63 ± 0.23	2.97 ± 0.15	6.27 ± 0.18	2.96 ± 0.14	2.89 ± 0.16	5.85 ± 0.24	2.61 ± 0.22

B. Calcium distribution

Ca^{2+} mg g-1	1			75			150		
Tissue	C	P	AM	C	P	AM	C	P	AM
SU	0.43 ± 0.13	0.64 ± 0.19	0.79 ± 0.05	0.41 ± 0.14	0.47 ± 0.23	0.24 ± 0.07	0.40 ± 0.14	0.59 ± 0.16	0.40 ± 0.12
YL	1.07 ± 0.29	1.06 ± 0.33	0.68 ± 0.16	0.92 ± 0.42	0.28 ± 0.07	0.12 ± 0.08	0.64 ± 0.38	0.56 ± 0.30	0.70 ± 0.17
ML	2.07 ± 0.57	2.37 ± 0.49	1.78 ± 0.61	2.12 ± 1.30	1.26 ± 0.21	1.24 ± 0.07	2.44 ± 0.57	1.89 ± 0.38	2.31 ± 0.43
OL	7.35 ± 0.79	6.11 ± 0.92	5.85 ± 0.48	6.16 ± 1.26	4.58 ± 0.98	6.38 ± 1.28	5.58 ± 0.92	5.59 ± 0.75	4.36 ± 0.96
SD	0.10 ± 0.08	0.44 ± 0.13	1.35 ±	0.09 ± 0.03	0.20 ± 0.07	0.15 ±	0.20 ± 0.05	0.19 ± 0.05	0.94 ±
TR	0.90 ± 0.17	0.63 ± 0.20	0.70 ± 0.17	0.47 ± 0.16	0.65 ± 0.11	0.29 ± 0.14	0.60 ± 0.06	0.90 ±	0.83 ± 0.11
FR	1.13 ± 0.13	1.03 ± 0.11	2.19 ± 0.48	0.80 ± 0.15	0.38 ± 0.14	1.18 ± 0.20	1.06 ± 0.29	0.43 ± 0.12	0.84 ± 0.23

C. Magnesium distribution

Mg^{2+} mg g-1	1			75			150		
Tissue	C	P	AM	C	P	AM	C	P	AM
SU	1.12 ± 0.10	1.39 ± 0.04	1.13 ± 0.06	1.10 ± 0.09	1.17 ± 0.12	1.32 ± 0.09	1.03 ± 0.18	1.21 ± 0.17	1.19 ± 0.11
YL	0.89 ± 0.04	1.18 ± 0.07	0.73 ± 0.05	0.95 ± 0.11	0.75 ± 0.05	0.74 ± 0.03	0.85 ± 0.09	0.61 ± 0.04	0.87 ± 0.09
ML	1.86 ± 0.34	1.97 ± 0.24	1.85 ± 0.31	1.89 ± 0.85	1.61 ± 0.33	1.48 ± 0.14	2.47 ± 0.48	2.23 ± 0.36	2.05 ± 0.54
OL	5.18 ± 0.42	5.48 ± 0.28	4.42 ± 0.54	4.76 ± 0.69	4.07 ± 0.53	5.29 ± 0.70	4.47 ± 0.47	4.03 ± 0.63	4.27 ± 0.87
SD	0.45 ± 0.07	0.48 ± 0.03	0.70 ± 0.34	0.37 ± 0.05	0.53 ± 0.07	0.46 ± 0.03	0.42 ± 0.04	0.45 ± 0.05	0.48 ± 0.04
TR	0.57 ± 0.06	0.95 ± 0.03	0.90 ± 0.03	0.50 ± 0.07	0.86 ± 0.05	0.69 ± 0.10	0.61 ± 0.05	0.69 ± 0.05	0.60 ± 0.09
FR	1.34 ± 0.08	1.36 ± 0.07	1.80 ± 0.23	1.05 ± 0.06	1.28 ± 0.07	1.23 ± 0.09	1.11 ± 0.10	1.39 ± 0.09	1.08 ± 0.15

D. Silicon distribution

Si mg g^{-1}	1			75			150		
Tissue	C	P	AM	C	P	AM	C	P	AM
SU	2.5 ± 0.19	2.36 ± 0.19	2.26 ± 0.28	2.50 ± 0.29	3.20 ± 0.52	3.16 ± 0.48	3.18 ± 0.43	4.81 ± 0.41	4.19 ± 0.41
YL	1.42 ± 0.46	1.33 ± 0.21	1.31 ± 0.19	1.83 ± 0.24	1.62 ± 0.15	1.85 ± 0.19	2.33 ± 0.33	2.01 ± 0.20	1.76 ± 0.15
ML	2.15 ± 0.23	1.78 ± 0.20	2.30 ± 0.27	2.40 ± 0.61	2.54 ± 0.46	2.51 ± 0.32	3.00 ± 0.42	4.00 ± 0.37	2.45 ± 0.41
OL	5.77 ± 0.83	4.72 ± 0.71	4.23 ± 0.48	5.97 ± 1.47	5.07 ± 1.12	6.36 ± 1.14	5.39 ± 0.78	5.75 ± 0.90	5.18 ± 1.28
SD	1.69 ± 0.11	1.88 ± 0.14	1.65 ± 0.29	1.61 ± 0.15	2.14 ± 0.31	1.99 ± 0.19	1.69 ± 0.05	2.17 ± 0.18	1.76 ± 0.04
TR	9.89 ± 3.18	10.7 ± 1.69	14.2 ± 3.20	9.07 ± 2.24	6.93 ± 1.15	14.1 ± 2.65	6.37 ± 1.20	7.40 ± 0.97	8.53 ± 1.39
FR	24.6 ± 9.07	25.1 ± 2.31	31.1 ± 3.22	12.8 ± 5.14	20.1 ± 5.30	26.7 ± 5.93	17.7 ± 3.51	13.3 ± 1.53	28.4 ± 6.80

Nutrient concentration (mg g^{-1}) was not affected by NaCl addition, thus C, P and AM plants showed the same values under 1, 75 and 150 mM NaCl (data not shown). Pi concentration was always higher in P plants than in C and AM ones, due to it higher concentrations in nutritional solution apply to P plants. Nonetheless, salinity stress was not affect P concentrations in any case.

As it was expected, Na^+ concentration increased in the salt treatments respect 1 mM NaCl in all plants, however, under 150 mM NaCl, AM plants showed low Na^+ concentration compared with C and P plants ($p = 0.0002$).
